# Prevalence of dual contraceptive use and associated factors among HIV positive women at University of Gondar Hospital, Northwest Ethiopia

**DOI:** 10.1186/s13104-019-4053-2

**Published:** 2019-01-18

**Authors:** Mebratu Mitiku Reta, Gizachew Assefa Tessema, Getachew Shiferaw

**Affiliations:** 1 Department of Internal Medicine, School of Medicine, University of Gondar, Gondar, Ethiopia; 2 Department of Reproductive Health, Institute of Public Health, University of Gondar, Gondar, Ethiopia; 3 Department of Gynecology and Obstetrics, School of Medicine, University of Gondar, Gondar, Ethiopia

**Keywords:** Dual contraceptive, HIV/AIDS, Women, Ethiopia

## Abstract

**Objective:**

Dual contraceptive is the use of male condom besides any modern contraceptive. It reduces parent to child transmission of HIV and other sexually transmitted infections between partners. The aim of this study was to investigate the prevalence and associated factors of dual contraceptive use among HIV positive women at University of Gondar Hospital, North West Ethiopia.

**Result:**

The prevalence of dual contraceptive use was found to be 13.2% (95% CI 10.5, 16.0). Partner involvement in post-test counseling [AOR = 3.11 (95% CI = 1.74, 5.57)], open partner discussion on using dual contraceptive [AOR = 7.84, 95% CI (4.26, 14.42)], provision of counseling about dual contraception [AOR = 6.56, 95% CI (3.54, 12.18)], age 18–24 years [AOR = 4.79, 95% CI (1.72, 13.32)], age 25–34 years [AOR = 1.97, 95% CI (1.01, 3.85)] and being a housewife [AOR = 4.38, 95% CI (1.89, 10.16)] were significant factors associated with dual contraceptive use. The prevalence of dual contraceptive use was low. This shows, there is a need to in promote partner involvement in HIV testing and counseling by offering counseling session in a couple-basis. It is also necessary for programmers to routinely focus on provision of dual contraception for HIV-infected women and Integration of family planning into HIV care follow-up clinic need to be strengthened.

## Introduction

In 2017, about 37 million people were living with HIV/AIDS in the world. More than two-third of them were living in sub-Saharan Africa and close to three-fourth of all AIDS related deaths were occurred in this region [1, 2]. Nearly 92% of pregnant women living with HIV were also from sub-Saharan Africa [3]. According to the 2011 Ethiopian demographic Health Survey (EDHS), the prevalence of HIV infection in women was 1.9% [4]. Dual contraceptive can reduce the transmission of resistant strains of HIV between partners [5]. Prevention of unintended pregnancy is the second approach in prevention of mother to child transmission of HIV (PMTCT) [6]. In doing so, contraceptive use is necessary and suggested by WHO and ministry of Health [7]. However, although HIV-positive women can use most of the available contraceptive methods, including hormonal, intrauterine devices and sterilization method [8], these methods are falling short in preventing transmission of HIV and STIs between partners. The effectiveness of estrogen based hormonal contraceptive is also affected by an interaction with some Antiretroviral (ARV) drugs such as Non Nucleotide reverse transcriptase inhibitors (NNRTIs) and protease inhibitors (PIs) [9]. As a result, an HIV-infected woman requires barrier methods such as, condom on top of the methods they used for the prevention of unintended pregnancies. Dual protection by using condom and any modern contraceptive method has paramount benefit for HIV positive women. Therefore, the study can provide evidence for the planners, officers and care providers in order to design appropriate interventions.

## Main text

### Methods

#### Study deign and participants

An institution based cross-sectional study was conducted at the University of Gondar Hospital, Northwest Ethiopia. The Hospital is located in Gondar town, 737 km from Addis Ababa. The hospital is serving for more than five million people in the catchment area. It has more than 600 inpatient admissions. There were a total of 6232 HIV patients at ART follow up clinic during data collection. The number of HIV positive women attending HIV care was 3219. HIV positive married women attending chronic HIV care follow up at the ART clinic during data collection were participated in the study. Pregnant HIV positive women were excluded from the study.

#### Sampling and sample size calculation

The sample size was determined using single population proportion formula by considering the following assumptions. Prevalence (P) was taken as 50%, since there was no study in Ethiopia. The 95% confidence interval, 4% level of precision and 5% non-response rate was considered to get a sample size of 630. Simple random sampling technique was used to select the study participants from July to August 2014.

#### Data collection techniques and procedures

Face to face interviewer administered questionnaire containing socio demographic and clinical characteristics and information on contraceptive use was used for data collection. Participants chart was also reviewed to record their date of HIV diagnosis, WHO clinical stage and current CD4 count. Once the participant interviewed, chart was labeled using a code to avoid repetition. The questionnaire was prepared in English and translated to Amharic and then back to English to maintain its consistency. Four trained nurses having a bachelor degree and one supervisor were involved in the data collection.

#### Data analysis

The filled questionnaires were checked for completeness, cleaned manually, pre-coded and entered to EPI Info version 3.5.3 statistical software before exported to SPSS version 20 for analysis. Descriptive and summary statistics were used to explain the study population with respect to the associated factors. Binary logistic regression was used primarily to check factors associated with the outcome variable and variables having p value of less than 0.2 were fitted into the multivariable logistic regression model. Backward stepwise multiple logistic regression model was fitted to identify the determinants of dual contraceptive utilization practice. Crude odds ratio with 95% CI was computed. Adjusted odds ratios with 95% CI were calculated. Statistical significance was inferred at a *P* value of < 0.05.

### Results

A total of 619 women were participated in the study with a response rate of 98.25%. The majority (92.9%) were urban residents. The mean age of the respondents was 32.26 [SD 6.3] years. One Hundred seventy (27.6%) of women were house wives. Five hundred ninety-three (95.8%) of women received post test counseling service. Only 38.1% percent of HIV positive women had discussion with their health care provider about dual contraceptive. Majority (65%) of the study participants had a CD4 count between 201 and 350 cells/mm^3^ (Table 1).

**Table 1:** Scio-demographic and clinical characteristics of HIV positive women at University of Gondar referral Hospital, North west Ethiopia, 2014 (n = 619)

Variables	Category	Frequency	Dual contraceptive use	Percent
Residence	Urban	575	76	13.2
	Rural	44	6	13.6
Age in years	18–24	56	12	21.43
	25–34	341	52	15.25
	≥ 35	222	18	8.1
Religion	Orthodox	546	77	14.1
	Muslim	58	4	6.9
	Others^a^	15	1	6.6
Ethnicity	Amhara	560	76	13.6
	Kemant	25	2	8
	Tigre	26	4	15.4
	Others^b^	8	0	0.0
Educational status	Can’t read and write	139	25	18
	Read and write only	22	2	9.1
	Primary school	190	165	86.84
	Secondary school	195	171	87.7
	TVET/College/University	73	6	8.22
Occupation	House wife	171	40	23.4
	Merchant	153	20	13.1
	Daily laborer	118	12	10.2
	Government employee	177	10	5.65
Partner’s occupation	Merchant	151	18	11.9
	Daily laborer	150	26	17.3
	Farmer	96	10	10.42
	Government employee	196	27	13.8
	Others^c^	25	1	4.0
Duration of HIV follow up (months)	< 6	33	4	12.1
	6–24	109	11	10.1
	> 24	477	67	14.05
Received post test counseling	Yes	477	70	14.7
	No	142	12	8.45
Counseled by HCP about dual contraceptive use	Yes	236	65	27.54
	No	383	17	4.44
HIV status disclosed to partner	Yes	452	72	15.9
	No	167	10	6.0
Partner involved in posttest counseling	Yes	245	56	22.86
	No	374	26	6.95
Partners HIV status	Reactive	356	66	18.54
	Non reactive	19	1	5.3
	Unknown	244	15	6.15
WHO Clinical stage	I	153	27	17.65
	II	112	12	10.71
	III	282	36	12.77
	IV	72	7	9.72
Recent CD4 count (cells/mm^3^)	< 200	84	5	5.95
	201–350	207	31	15
	> 350	328	46	14

^a^Catholic, Wakefata, ^b^ Oromo, Gurage, ^c^ Jobless, private employee

Five hundred twenty-five (84.8%) women had ever given birth. One hundred thirteen (18.3%) and 36 (5.8%) had history of abortion and still birth respectively. Six hundred four (97.6%) of the respondents were informed about modern contraceptive methods. Depo-Provera, Oral contraceptive pills and male condom were the most recognized family planning methods by the women (92.9%, 91.1% and 84.3%) respectively. Two hundred forty-one (38.9%) heard about dual contraceptive methods. About (38.1% and 33.9%) of the study participants had discussion with health care worker and with their partner about the use of dual contraceptives respectively. Male condom was the most commonly used contraceptive by 33.12% of their partners (Table 2).

**Table 2:** Information on modern contraceptive methods among HIV positive women at University of Gondar referral Hospital, North west Ethiopia, 2014 (n = 619)

Variables	Frequency	Percentage
*Informed regarding modern contraceptive methods*		
OCP	564	91.1
IUCD	443	71.6
Depo provera	575	92.9
Norplant	446	72.1
Female condom	122	19.7
Female sterilization	94	15.2
Male condom	522	84.3
Male sterilization	84	13.6
Emergency pills	128	20.7
Ever heard about modern contraceptive	606	97.9
Ever heard about dual contraceptive	241	38.9
Counseled about dual contraceptive by HCP	236	38.1
Discussed about dual contraceptive with partner/husband	210	33.9

Two hundred sixty-two (42.3%) respondents were current users of modern contraceptive methods. Male condoms were the most commonly used method. In this study the prevalence of dual contraceptive was 13.2% [95% CI (10.5, 16.0)]. Among those who were using the dual contraceptives, majority of them used condom with injectable 57 (69.5%). About 66% of them were using condom consistently. Fear of side effects, partner disapproval to use condom and lack of women’s self-interest to use condom were major reasons not to use dual contraceptive. However, they were not statistically significant Multivariable logistic regression indicated that, partner involvement in post test counseling, open discussion between partners on dual contraceptive, counseling by health care providers (HCP) about its benefit, Age, and being a housewife were significant factors associated with dual contraceptive use. Partner involvement in post test counseling had three times more odds of dual contraceptive utilization than those who did not have any partner involvement [AOR = 3.11 (95% CI = 1.74, 5.57)]. Those who had Open partner discussion about dual contraceptive use were almost 8 times more odds of dual contraceptive use than who never discussed [AOR = 7.84, 95% CI (4.26, 14.42)]. Women counseled by health care provider to use dual contraceptive were 7 times more odds to use [AOR = 6.56, 95% CI (3.54, 12.18)]. Age 18–24 years were close to five times more odds of using dual contraceptive [AOR = 4.79, 95% CI (1.72, 13.32)] and those age 25–34 years had two times more odds of dual contraceptive use than age 35 and above [AOR = 1.97, 95% CI (1.01, 3.85)]. Housewives were 4.4 times more odds of using dual contraceptive than government employees [AOR = 4.38, 95% CI (1.89, 10.16)] (Table 3).

**Table 3:** Logistic regression analysis of variables on dual contraceptive practice among HIV positive women at University of Gondar referral Hospital, North west Ethiopia, 2014 (n = 619)

Variables	Categories	Dual contraceptive use (n = 619)		COR (95% CI)	AOR (95% CI)	P value
		Yes	No			
Age in years	18–24	12	44	3.1 (1.39, 6.88)	4.79 (1.72, 13.32)	0.003
	25–34	52	29	2.04 (1.16, 3.59)	1.97 (1.01, 3.85)	0.048
	≥ 35	18	204	1	1	
Occupation	Housewife	40	131	5.1 (2.46, 10.56)	4.38 (1.89, 10.16)	0.001
	Merchant	20	133	2.51 (1.14, 5.55)	1.99 (0.82, 4.88)	
	Daily laborer	12	106	1.89 (0.79, 4.53	1.43 (0.54, 3.87)	
	Government employee	10	167	1	1	
Counseled by HCP about dual contraceptive	Yes	65	171	8.18 (4.66, 14.38)	6.56 (3.54, 12.18)	< 0.001
	No	17	366	1	1	
HIV status disclosed to partner	Yes	72	380	2.98 (1.50, 5.91)	1.12 (0.48, 2.58)	
	No	10	157	1	1	
Partner involved in post test counseling	Yes	56	189	3.97 (2.41, 6.52)	3.11 (1.74, 5.57)	< 0.001
	No	26	348	1	1	
Ever heard about dual contraceptive	Yes	82	524	7.84 (4.46, 13.78)	1.19 (0.10, 14.33)	
	No	0	13	1	1	
Discussed with partner about dual contraceptive	Yes	60	150	7.04 (4.17–11.88)	7.84 (4.26, 14.42)	< 0.001
	No	22	387	1	1	
Ever gave birth	Yes	74	451	1.76 (0.82, 3.79)	1.51 (0.58, 3.96)	
	No	8	86	1	1	
Most recent CD4 count (cells/mm^3^)	< 200	5	79	1		
	200–350	31	176	2.76 (1.04, 7.42)	1.57 (0.51, 4.80)	
	≥ 350	46	282	2.58 (0.99, 6.71)	1.19 (0.40, 3.52	

### Discussion

The use of dual contraceptive is very important for effectiveness of PMTCT program. However, this study revealed that the prevalence of dual contraceptive utilization was low. Similar result was also reported in Kenya (13.5%) [10], Uganda (17%) [11], Uganda (17%) [12], Zambia (17%) [13]. This may be due you to the variation in time of enrolment in HIV care and the time that the study was conducted (16). As compared to the study in Ireland (22%) [14], Nigeria (25.1%) [15], Zambia (25%) [16], India (23%) [16], South Africa (22%) [17], Southwest Ethiopia (19.8%) [18], Thailand (29.6%) [19], Tanzania (33%) [20], Kenya [21] and Fitche hospital, Ethiopia (32%) [22] our finding is lower. This finding was also much lower than the finding of the study done in Nigeria and United States of America where (50% and 59.7%) of HIV positive women were using dual contraceptive methods respectively [23, 24]. This may be due to the fact that women in the study area may lack negotiation skill as a result of socio-cultural influence that hinders open partner discussion about contraceptive. It may also be due to lack of knowledge about dual contraceptive when compared with women in Ireland, India, South Africa and Nigeria.

Concerning the factors affecting dual contraceptive methods utilization, partner involvement in HIV testing and counseling was found to be significant factor to use dual contraceptive methods by HIV positive women. Those who were tested and counseled together with their partner were 3.1 times more odds to use dual contraceptive [AOR = 3.11 (95% CI = 1.74, 5.57)]. This finding is supported by a survey conducted in Addis Ababa [25] and India [26]. This suggests that testing and counseling partners together encourages them to have open discussion while choosing the appropriate contraceptive methods they use. An open partner discussion on choosing contraceptive was significantly associated with dual contraceptive use. Those who ever had open discussion were almost 8 times more odds of dual contraceptive use than who never discussed [AOR = 7.84, 95% CI (4.26, 14.42)]. This was again supported by a study conducted in Addis Ababa [25], Tanzania [20], Bahir Dar [27], south west Ethiopia [28] and Tigray region [29]. This suggests that, creating an open discussion between partners about contraceptive choice and its benefit is advantageous. Counseling by health care provider was significant factor to use dual contraceptive among HIV positive women. Those counseled by health care provider to use dual contraceptive were six and half times more odds of using dual contraceptive [AOR = 6.56, 95% CI (3.54, 12.18)]. This agrees with a study conducted in Lusaka, Zambia [13] and India [26], Tanzania [20], Kenya [21] and Bahir Dar [27]. This strongly indicates that counseling of HIV positive women can improve the utilization of dual contraceptive use in preventing unintended pregnancy and sexually transmitted infections. It also suggests that health care providers need to focus on counseling on dual contraceptives and its benefit to HIV positive women attending care at ART clinic. This study also showed that age of the respondents was one of the important factors. Those women age 18–24 years [AOR = 4.79, 95% CI (1.72, 13.32)], age 25–34 years [AOR = 1.97, 95% CI (1.01, 3.85)] were having more odds of dual contraceptive use than those above 35 years old. This was supported by the finding of the study in Northern Uganda [30]. This finding was also supported by the finding in a systematic review done by Marge Berer [31]. It may be due to the fact that those young women may not be interested to have child at this age and may have better understanding on the benefit of using dual contraceptive. However, the finding of this study was not consistent with the finding of the study conducted in southwest Ethiopia [28]. This variation may be due to the fact that the difference in the study design and sample size; mixed method was used in the study conducted in southwest Ethiopia with small sample size. Being a house wife was significantly associated with dual contraceptive use. Those housewife in their occupation were about 4.5 times more odds of using dual contraceptive compared to government employees [AOR = 4.38, 95% CI (1.89, 10.16)]. The reason for this may be due to economical concern. Housewives do not have their own monthly income and they are dependent on their partner. Due to this, they may believe that they can’t afford expenses if they become pregnant and give birth. This assumption is supported by the finding of a study conducted northern Uganda [30] and Southwestern Uganda [32].

### Conclusion

Only small proportion of HIV positive women were using dual contraceptive. Partner involvement in post test counseling, open partner discussion about dual contraceptive, providing counseling by HCP about dual contraceptive, age and occupation of the women were positively associated with dual contraceptive use. Simultaneous HIV testing and counseling of women with their partner, open partner discussion s on family planning choice and provision of counseling by health care providers shall be encouraged.

## Limitations

Major limitations in this study are absence of variables that might have been associated with dual contraceptive use, such as: social support and variables related to mental health. Absence of partner involvement in the study was also another limitation. A cross-sectional study design lacks temporal association. In addition, since the questionnaire covers private issues there might be information bias.

## Abbreviations

AIDS: Acquired Immune deficiency Syndrome
ART: antiretroviral therapy
ARV: antiretroviral
EDHS: Ethiopian Demographic Health Survey
HCP: Health Care Provider
HCW: health care worker
PMTCT: Prevention of Mother to Child Transmission
STIs: Sexually Transmitted Infections
TVET: Technical and Vocational Education and Training
WHO: World Health Organization
